# Comparison of local activation, functional connectivity, and structural connectivity in the N-back task

**DOI:** 10.3389/fnins.2024.1337976

**Published:** 2024-03-04

**Authors:** Takatoshi Satake, Ai Taki, Kazumi Kasahara, Daisuke Yoshimaru, Tomokazu Tsurugizawa

**Affiliations:** ^1^Human Informatics and Interaction Research Institute, National Institute of Advanced Industrial Science and Technology (AIST), Ibaraki, Japan; ^2^Graduate School of Comprehensive Human Science, University of Tsukuba, Tsukuba, Japan; ^3^Faculty of Engineering, Information and Systems, University of Tsukuba, Tsukuba, Japan; ^4^Division of Regenerative Medicine, The Jikei University School of Medicine, Tokyo, Japan

**Keywords:** functional MRI, diffusion tensor imaging, N-back task, structural connectivity, local activation

## Abstract

The N-back task is widely used to investigate working memory. Previous functional magnetic resonance imaging (fMRI) studies have shown that local brain activation depends on the difficulty of the N-back task. Recently, changes in functional connectivity and local activation during a task, such as a single-hand movement task, have been reported to give the distinct information. However, previous studies have not investigated functional connectivity changes in the entire brain during N-back tasks. In this study, we compared alterations in functional connectivity and local activation related to the difficulty of the N-back task. Because structural connectivity has been reported to be associated with local activation, we also investigated the relationship between structural connectivity and accuracy in a N-back task using diffusion tensor imaging (DTI). Changes in functional connectivity depend on the difficulty of the N-back task in a manner different from local activation, and the 2-back task is the best method for investigating working memory. This indicates that local activation and functional connectivity reflect different neuronal events during the N-back task. The top 10 structural connectivities associated with accuracy in the 2-back task were locally activated during the 2-back task. Therefore, structural connectivity as well as fMRI will be useful for predicting the accuracy of the 2-back task.

## Introduction

Working memory is essential for the cognitive process that keeps small amounts of information active for later recall and manipulates limited amounts of information for current computation (Daneman, 1980; Baddeley, 1992). Working memory capacity is the amount of information that can be retained in mind for a short period. The amount of accessible information is determined by working memory capacity, which depends on the individual (Wilhelm et al., 2013). The n-back task, a continuous-recognition measure that uses sequences of stimuli, such as letters, is a simple tool for monitoring working memory processes (Kirchner, 1958). For this task, the participants were required to monitor a series of stimuli and respond whenever a stimulus was presented, the same as in the N trials. The difficulty of the n-back task varies with N (i.e., the 3-back task is more complicated than the 1-back task) (Picchioni et al., 2007; Lamichhane et al., 2020). Previous studies using functional magnetic resonance imaging (fMRI), which detects blood oxygenation level-dependent (BOLD) responses coupled with neuronal activation, have revealed the locations of local activation for N-back tasks. In general, the BOLD response is calculated using a general linear model (GLM), which is a model of the temporal convolution of the hemodynamic response function to the task period. Owen et al. reported local activation in frontoparietal regions, including the ventrolateral, dorsolateral, and frontopolar prefrontal cortices, in addition to the dorsal cingulate and premotor cortices, during an N-back task (Owen et al., 2005). Another meta-analysis revealed the largest clusters in the prefrontal and parietal cortices of the left hemisphere, including the middle frontal gyrus (MFG) and inferior parietal lobule, in young adults during an N-back task. Other areas include the medial frontal gyrus, insula, nuclei of the basal ganglia, and cerebellum (Yaple et al., 2019). Several studies have evaluated the effect of the N-back task difficulty by changing the number of N in the N-back task on the BOLD responses in the prefrontal and posterior parietal cortices. The amplitudes of the evoked responses in the frontal and parietal cortices increased as the number increased from 1- to 3-back (Braver et al., 1997; Jaeggi et al., 2003). Another study explored the N-back task, with scores ranging from *N* = 1 to *N* = 6. The BOLD response of the lateral prefrontal cortex was found to increase from 1-back to 3-back and then plateau until 6-back (Lamichhane et al., 2020). These results indicate that the difficulty of N-back task affects the degree of working memory processing in the brain.

These previous studies investigated local activation during the N-back task but did not assess functional connectivity. The functional connectivity is derived from the synchronization of neuronal oscillations between anatomically separated regions. Importantly, functional connectivity is related to cognitive function (Power et al., 2011). The generalized psychophysiological interaction (gPPI) is used to investigate functional connectivity during cognitive tasks (McLaren et al., 2012). GLM analysis indicates local neuronal activation, whereas gPPI showed a change in the functional connectivity between the affected brain regions during the task. This approach enables us to investigate functional connectivity, which is more sensitive and specific to the task phase (Tsurugizawa et al., 2023).

In addition to fMRI, the white matter microstructure, which can be estimated using diffusion tensor imaging (DTI), is essential for cognitive processing and memory. Several DTI studies on working memory tasks have revealed positive correlations between fractional anisotropy in the frontoparietal white matter and working memory task performance in children (Olesen et al., 2003; Klingberg, 2006; Darki and Klingberg, 2015) and adults (Schulze et al., 2011). The anatomical connections of the white matter between anatomically separated regions, called structural connectivity, can be calculated using DTI. Previous studies have suggested its use in predicting working memory (McKenna et al., 2015). However, to date, no study has compared the structural connectivity, functional connectivity, and local activation in the N-back task.

In the present study, we compared the functional connectivity, structural connectivity, and local activation during an N-back task. We used GLM and gPPI analyses to investigate local activation and functional connectivity depending on the difficulty of the N-back task. Because we found that the 2-back task was better for investigating working memory from behavior, the functional and structural connectivity associated with accuracy were also investigated.

## Materials and methods

### Participants

Twenty-five healthy young adults (14 males and 11 females; age 24.3 ± 7.32 years) participated in the fMRI experiment. All the participants were right-handed and had no history of alcohol abuse, neurological diseases, or learning disabilities. All experimental procedures and protocols were approved by the Institutional Review Board of the National Institute of Advanced Industrial Science and Technology.

### Behavioral analysis

R Statistical Software (v4.3.1, R Core Team, 2023), RStudio (2023.06.1, Rstudio Team, 2023), Coin [v1.4.2, (Hothorn et al., 2006)] and Tidyverse [v2.0.0, (Wickham et al., 2019)] were used for statistical behavioral analyses.

### fMRI N-back task and acquisition parameters

Participants were scanned with a 32-channel phased array receiving head coil (3.0 T scanner, Philips, Netherlands). The participants wore earplugs to reduce scanner noise, and foam padding was used to suppress head motion. T1-weighted 3D magnetization prepared-rapid Gradient Echo (MPRAGE) images were obtained using the following parameters: repetition time (TR) = 11 ms, echo time (TE) = 5.1 ms, flip angle = 8°, matrix = 368 × 315 × 257, and resolution = 0.7 × 0.76 × 0.7 mm^3^/voxel. The fMRI data were acquired using gradient echo Echo-Planar Imaging (EPI) with the following conditions: TR = 1,500 ms, TE = 30 ms, flip angle = 80°, multiband = 2, matrix = 76 × 76 × 44, resolution = 2.5 × 2.5 × 2.5 mm^3^/voxel. The fMRI data for each task were acquired for 8 min 45 s (350 volumes), and the resting-state fMRI data were acquired for 10 min 30 s (420 volumes).

Following the fMRI experiment, DTI data were acquired with diffusion-weighted spin echo EPI and the following parameters: TR = 7,263 ms, TE = 95 ms, matrix = 112 × 110 × 55, resolution = 2.0 × 2.0 × 2.0 mm^3^/voxel with 1 b = 0 s/mm^2^ image and with 64 diffusion directions defined evenly across the sphere with a diffusion weighting of b = 1,000 s/mm^2^.

### N-back task with fMRI

**Figure 1:**
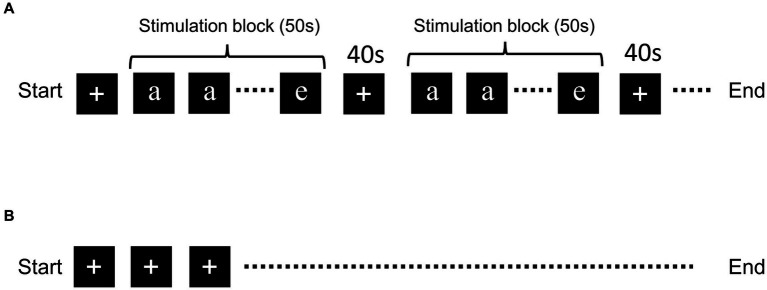
Schematic of the N-back task and resting-state fMRI. Schematic representation of the **(A)** N-back task and **(B)** resting-state fMRI. Five letters (“a,” “b,” “c,” “d,” and “e”) were presented randomly during the stimulation block. Letter presentation was repeated 20 times for each block. The “+” mark was presented during the resting period.

The N-back task was performed with five letters: “a,” “b,” “c,” “d,” and “e” (Figure 1). The letters were randomly presented on a computer screen located outside the MRI bore and the participants viewed the letters through a tilted mirror. The tasks were performed in the order 1- of 3-back task. Each of the letters was presented for 2.5 s, twenty times in each stimulation block (in total 50 s), and the “+” mark was presented for the 40-s resting period (Figure 1). Five stimulation blocks were used in each experiment. When participants recognized that the current letter was the same as the previous letter, they pushed the button. The timing of the alphabet presentation and data collection was determined using PsychoPy.^1^ fMRI scanning with the “+” mark throughout MRI scan was performed as the negative control (Kumari et al., 2006; Yaple et al., 2021).

### Preprocessing of the fMRI data

Statistical parametric mapping SPM12 software (Welcome Trust Center for Neuroimaging, United Kingdom) was used in the preprocessing steps, including slice timing correction, motion correction by realignment, normalization, and smoothing with a Gaussian filter (8 × 8 × 8 mm^2^/voxel of half-width at half-maximum). Following the preprocessing with SPM12, fMRI data were detrended, and slow periodic fluctuations were extracted using a bandpass filter (0.008–0.09 Hz) with CONN toolbox.^2^

### General linear model (GLM) analysis

For the first level (fixed-effect analysis), an onset regressor defined the onset of the N-back task, and the block length was set to 50 s for each session. The hemodynamic response was modeled using the canonical hemodynamic response function in SPM12. Six head-movement parameters estimated during the realignment process were used as regressors to determine the effect of head motion. The resulting contrast images were calculated by setting the targeting regressor (regressor of the N-back task) to 1 and the others to zero in N-back tasks and no N-back task. The calculated contrasts were then used for a second-level analysis (random effect analysis). The contrasts of 1, 2, and 3-back were compared with no N-back contrast. A high-pass filter of 1/128 Hz was used to remove this detrending. The contrasts of the 1-, 2-, and 3-back tasks were compared with the no N-back contrast. Model estimation was performed using a paired t-test to compare each task with the negative control. The significant voxels were thresholded at *p* < 0.05, and family-wise error (FWE) was corrected with a cluster size. The activated regions were determined by counting the voxels in each region of interest (ROI). To investigate the correlation between fMRI beta values and task performance, the beta values of individuals within ROI were extracted from the contrast image that were calculated in first level analysis and were averaged.

### ROI-To-ROI gPPI analysis

The ROI-to-ROI gPPI was performed using the CONN toolbox to assess the brain regions that interact in a task-dependent manner. A total of 132 ROIs from the automated anatomical labeling atlas were used for ROI analysis. ROI-to-ROI gPPI analysis was performed for every possible pairwise combination of the selected ROIs for each participant. The predictors included the estimated time course of the task (psychological term), time-series regressor of BOLD signal changes in each ROI (physiological term), and interactions between psychological and physiological terms (PPI term), which consisted of the product of the psychological term multiplied by the physiological term. For the first-level analysis, a PPI regressor (PPI term) was generated for each condition (resting and task) as the product of the ROI time series multiplied by the task effect, and the beta weight was calculated for all ROIs. A random effects analysis was used across participants at the group level, and a one-sample t-test was conducted to compare ROI-based connectivity at rest and during the tasks.

### DTI analysis

DTI data were preprocessed by denoising, eddy current correction, and motion correction using MRtrix3 (Tournier et al., 2019). The DTI data were denoised using the *dwidenoise* command. Eddy currents and motion corrections were performed using the *dwifslprepcroc* command (Ning et al., 2021). A b0 image was registered on a T1-weighted image. T1-weighted images were then registered onto the standard MNI space with a nonlinear transformation using FNIRT in FSL (University of Oxford, United Kingdom) (Park et al., 2015). Nonlinear registration of the native diffusion space to the MNI standard space was performed using these parameters. Structural connectivity was computed from preprocessed DTI data using the DSI Studio software (Tsurugizawa et al., 2020). The preprocessed DTI data were then used for fiber tracking. First, the differential tractogram was obtained by placing 1,000,000 seeding points in the white matter using the following parameters: QA threshold = 0.1, angular threshold = 45°, step size = 0.5 mm, minimum length = 1 mm, and maximum length = 300 mm (Bauer et al., 2017). The same ROIs as gPPI were used for brain parcellation, and the connectivity matrix was calculated using the connecting tracks. The connectivity matrix was calculated by calculating the number of tracts passing through the two ROIs. The number of fibers is proportional to the fiber length from a tracking algorithm when fibers are calculated between two ROIs. The number of tracts between two ROIs should be scaled by the fiber length to account for this effect (Latora and Marchiori, 2001; Cheng et al., 2012). The number of tracts between two ROIs should be scaled by the fiber length to account for this effect. This number was then normalized by multiplying it with the sum of the inverses of the lengths.

### Statistical tests

We performed simple regression analysis for task accuracy acquired during fMRI scanning as behavior analyses. Friedman non-parametric tests were used to compare task accuracy and response time between tasks. As a *post hoc* test, Wilcoxon signed-rank tests were conducted for each of the three combinations: 1-back vs. 2-back, 2-back vs. 3-back, and 1-back vs. 3-back.

We performed a paired t-test with Bonferroni correction (alpha significance of 0.05/3 = 0.017) on the accuracy and response time of the 1-, 2-, and 3-back tasks. For ROI-to-ROI gPPI, the statistical significance of ROI-ROI connectivity was assessed using a paired t-test or two-sample t-test with a corrected threshold of *p* < 0.05 and FDR-corrected using the CONN toolbox. The least-squares regression analysis between beta values, functional connectivity, structural connectivity, and accuracy of each task was performed.

## Results

### Task performance for the N-back task

**Figure 2:**
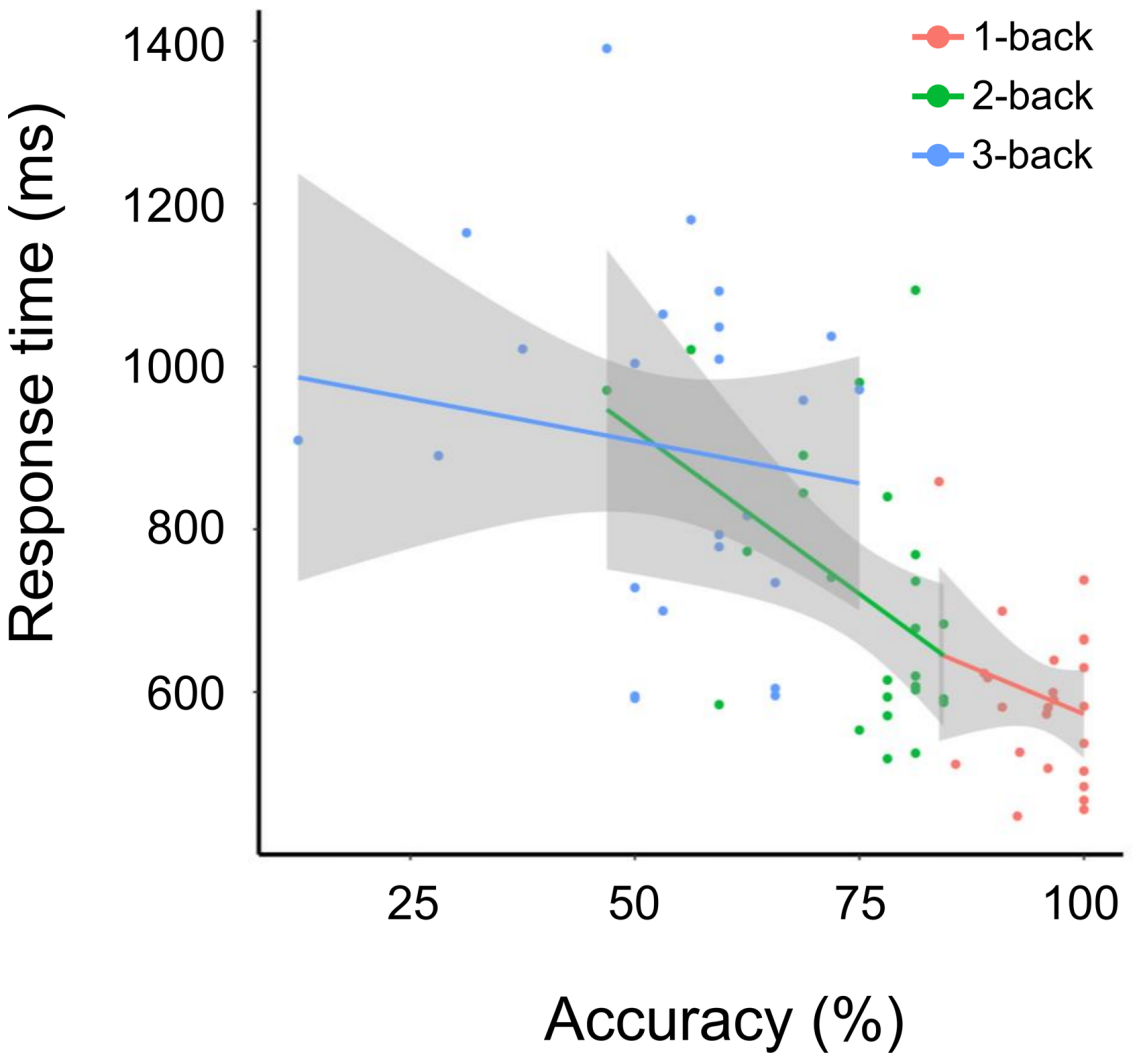
Accuracy and response time for the 1-, 2-, and 3-back tasks. Correlation between accuracy and response time (ms) for tasks 1-back (red), 2-back (green), and 3-back (blue). The solid line indicates fitting using the least-squares method.

Overall, accuracy negatively correlated with N-back task difficulty, whereas response time positively correlated with N-back task difficulty. The correlation between accuracy and response time was calculated using the least-squares method (R^2^ was 1.18 × 10^−2^ for 1-back, 0.18 for 2-back, and − 1.93 × 10^−2^ for 3-back) (Figure 2). The accuracies for 1-back, 2-back, and 3-back were 95.70 ± 4.98%, 75.12 ± 9.69%, and 52.75 ± 15.18%, respectively. The response times for 1-back, 2-back, and 3-back were 0.59 ± 0.09 s, 0.71 ± 0.17 s, and 0.90 ± 0.20 s, respectively. The 1-back task led to higher accuracy and shorter response time than the 2-back and 3-back tasks. Significant differences between groups were found for both response accuracy and reaction time (*p*-value = 1.39 × 10^−11^, chi-squared = 50 for accuracy, value of *p* = 7.12 × 10^−9^, chi-squared = 37.52 for response time, based on the Friedman’s test). Post-hoc analysis using the Wilcoxon test revealed significant differences for all 1-backs vs. 2-back (accuracy: *p*-value = 5.96 × 10^−8^, response time: *p*-value = 5.39 × 10^−5^), 2-back vs. 3-back (*p*-value = 5.96 × 10^−8^ for accuracy, *p*-value = 6.56 × 10^−6^ for response time), and 1-back vs. 3-back (*p*-value = 5.96 × 10^−8^ for accuracy, *p*-value = 1.29 × 10^−7^ for response time). These results were consistent with those of a previous study (Lamichhane et al., 2020).

### Altered functional connectivity during the N-back task

**Figure 3:**
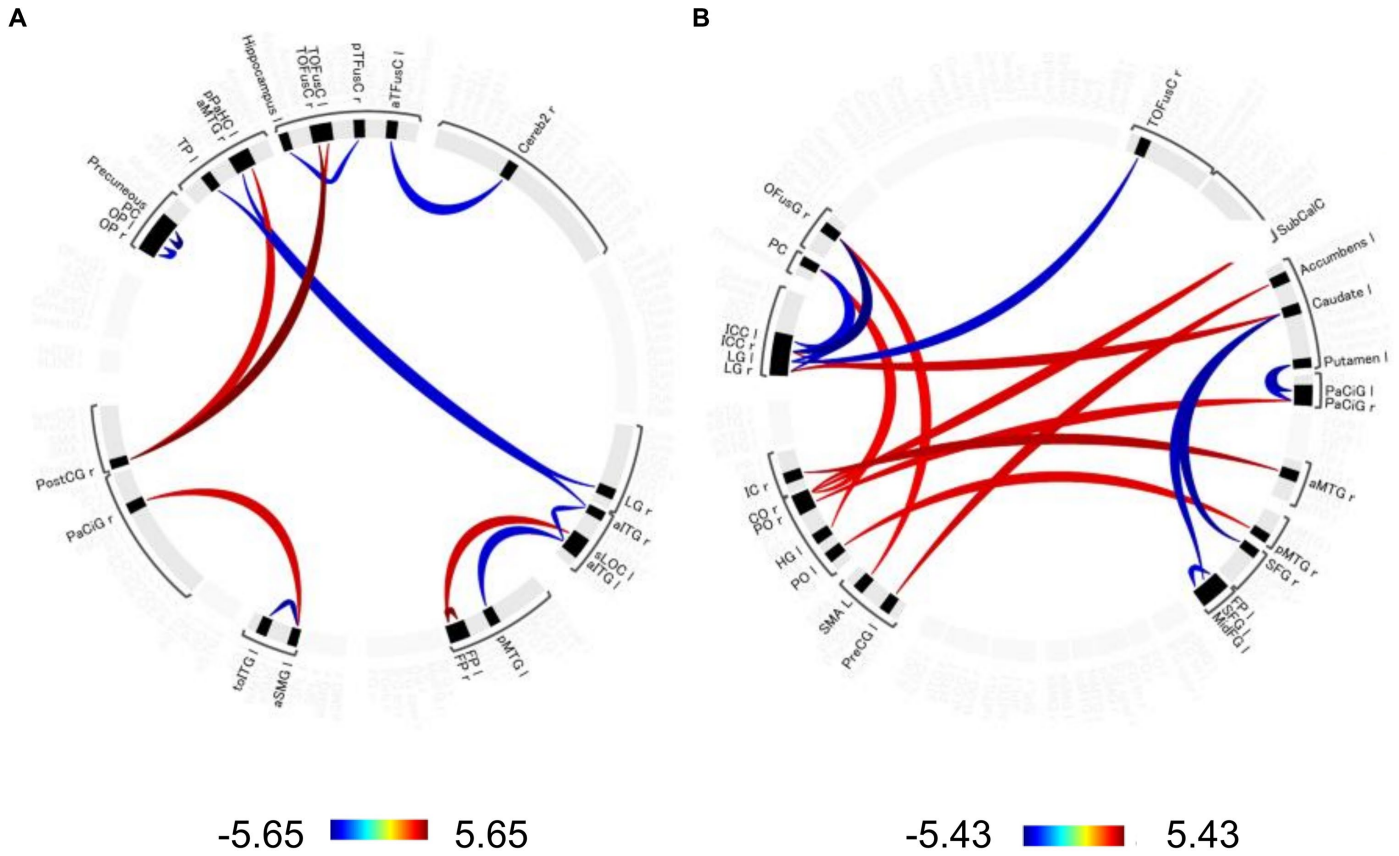
Significant changes in functional connectivity during the 2-and 3-back task. Circle plot of significant changes in functional connectivity for the **(A)** 2-back task and **(B)** 3-back task compared to the no-N-back task (*p* < 0.05, FDR-corrected). Positive color indicates an increased functional connectivity *T*-value. Negative colors indicate *T*-values of decreased functional connectivity. aTFusC, temporal fusiform cortex, anterior division; aITG, inferior temporal gyrus, anterior division; aMTG, middle temporal gyrus, anterior division; aSMG, supramarginal gyrus, anterior division; Cereb2, cerebelum crus2; CO, central opercular cortex; FP, frontal pole; HG, Heschl’s gyrus; IC, insular cortex; ICC, intracalcarine cortex; LG, lingual gyrus; MidFG, middle frontal gyrus; OFusG, occipital fusiform gyrus; OP, occipital pole; PaCiG, paracingulate gyrus; PC, cingulate gyrus, posterior division; pMTG, middle temporal gyrus, posterior division; PO, parietal operculum cortex; PostCG, postcentral gyrus; pPaHC, parahippocampal gyrus, posterior division; PreCG, precentral gyrus; pTFusC, temporal fusiform cortex, posterior division; SFG, superior frontal gyrus; sLOC, lateral occipital cortex, superior division; SMA, supplementary motor area; SubCalC, subcallosal cortex; TOFusC, temporal occipital fusiform cortex; toITG, inferior temporal gyrus, temporooccipital part; TP, temporal pole.

Significant changes in functional connectivity were observed in the 1-, 2-, and 3-back tasks (Figure 3). No significant changes in the functional connectivity were observed during the 1-back task. In contrast, functional connectivity in several brain regions changed differently in the 2-back and 3-back tasks. During the 2-back task, functional connectivity increased among several brain regions, including the left anterior supramaginal gyrus (aSMG), right paracingulate gyrus (PaCiG), bilateral frontal pole (FP), left superior lateral occipital cortex (sLOC), posterior cingulate gyrus (postCG), and bilateral temporal occipital fusiform cortex (ToFusC) (Figure 3A and Table 1). Functional connectivity decreased in several brain regions, including the right anterior inferior temporal gyrus (aITG), left temporal pole (TP), and bilateral occipital pole (OP) during the 2-back task compared with the rest period.

**Table 1:** The significant changes (*p* < 0.05, FDR-corrected) in functional connectivity for 2-back task.

Connectivity		*T*-value
Right temporal occipital fusiform cortex	Right postcentral gyrus	5.65
Right postcentral gyrus	Right temporal occipital	5.10
Left frontal pole	Fusiform cortex	5.50
Left superior lateral occipital cortex	Right frontal pole	4.72
Left supramarginal gyrus, anterior division	Right frontal pole	4.64
Right postcentral gyrus	Right paracingulate gyrus	4.64
Left temporal occipital fusiform cortex	Left parahippocampal gyrus, posterior division	4.59
Posterior cingulate gyrus	Precuneous cortex	−5.63
Left inferior temporal gyrus, temporooccipital part	Left supramarginal gyrus, anterior division	−5.10
Right inferior temporal gyrus, anterior division	Left temporal pole	−4.76
Left inferior temporal gyrus, anterior division	Left middle temporal gyrus, posterior division	−4.66
Left occipital pole	Right occipital pole	−4.62
Right inferior temporal gyrus, anterior division	Left inferior temporal gyrus, anterior division	−4.57
Right middle temporal gyrus, anterior division	Right lingual gyrus	−4.55
Left temporal fusiform cortex, anterior division	Right cerebelum crus2	−4.50
Left hippocampus	Right temporal fusiform cortex, posterior division	−4.49

The negative *T*-value indicates the decreased functional connectivity and positive *T*-value indicates the increased functional connectivity.

The 3-back task evoked significant changes in functional connectivity in distinct brain regions compared with the 2-back task. Functional connectivity increased significantly in the right central/parietal opercular cortex (CO/PO) and right insular cortex (IC) (Figure 3B and Table 2). In particular, the right IC and bilateral opercula showed increased functional connectivity with the right frontal lobe and cingulate cortex. Functional connectivity between the right anterior medial temporal gyrus (aMTG) and right IC, between the right posterior middle temporal gyrus (pMTG) and left PO, and between the right CO and subcallosal cortex (SubCalC) increased during the 3-back task. Regions related to motor regulation (the left precentral gyrus (preCG) and supplementary motor area (SMA)) showed increased functional connectivity with the left accumbens and right occipital fusiform gyrus (OFusG). The basal ganglia also exhibited decreased functional connectivity in several regions. Functional connectivity between the bilateral superior frontal gyrus (SFG) and left caudate and between the left putamen and bilateral PaCiG was significantly decreased. Functional connectivity was also significantly decreased between the visual cortex and occipital fusiform cortex, including the bilateral lingual gyrus (LG) and bilateral intracranial compliance (ICC).

**Table 2:** The significant changes (*p* < 0.05, FDR-corrected) in functional connectivity for 3-back task.

Connectivity		*T*-value
Right superior frontal gyrus	Left caudate	5.01
Right insular cortex	Right anterior middle temporal gyrus	4.82
Left caudate	Right lingual gyrus	4.54
Right central opercular cortex	Right anterior middle temporal gyrus	4.50
Left caudate	Right intracalcarine cortex	4.47
Left precentral gyrus	Left accumbens	4.46
Right central opercular cortex	Right subcallosal cortex	4.39
Right parietal operculum cortex	Right paracingulate gyrus	4.37
Left parietal operculum cortex	Right posterior middle temporal gyrus	4.31
Left supplementary motor area	Right occipital fusiform gyrus	4.19
Right central opercular cortex	Posterior cingulate gyrus	4.19
Left heschl’s gyrus	Posterior cingulate gyrus	4.18
Right occipital fusiform gyrus	Right intracalcarine cortex	−5.43
Left paracingulate gyrus	Left putamen	−4.89
Left superior frontal gyrus	Left caudate	−4.81
Right occipital fusiform gyrus	Right lingual gyrus	−4.70
Right temporal occipital fusiform cortex	Left lingual gyrus	−4.58
Left middle frontal gyrus	Left frontal pole	−4.50
Cingulate gyrus, posterior division	Right intracalcarine cortex	−4.43
Right occipital fusiform gyrus	Left intracalcarine cortex	−4.38
Right occipital fusiform gyrus	Left lingual gyrus	−4.35
Right paracingulate gyrus	Left putamen	−4.32

The negative *T*-value indicates the decreased functional connectivity and positive *T*-value indicates the increased functional connectivity.

### Bold response during the N-back task

**Figure 4:**
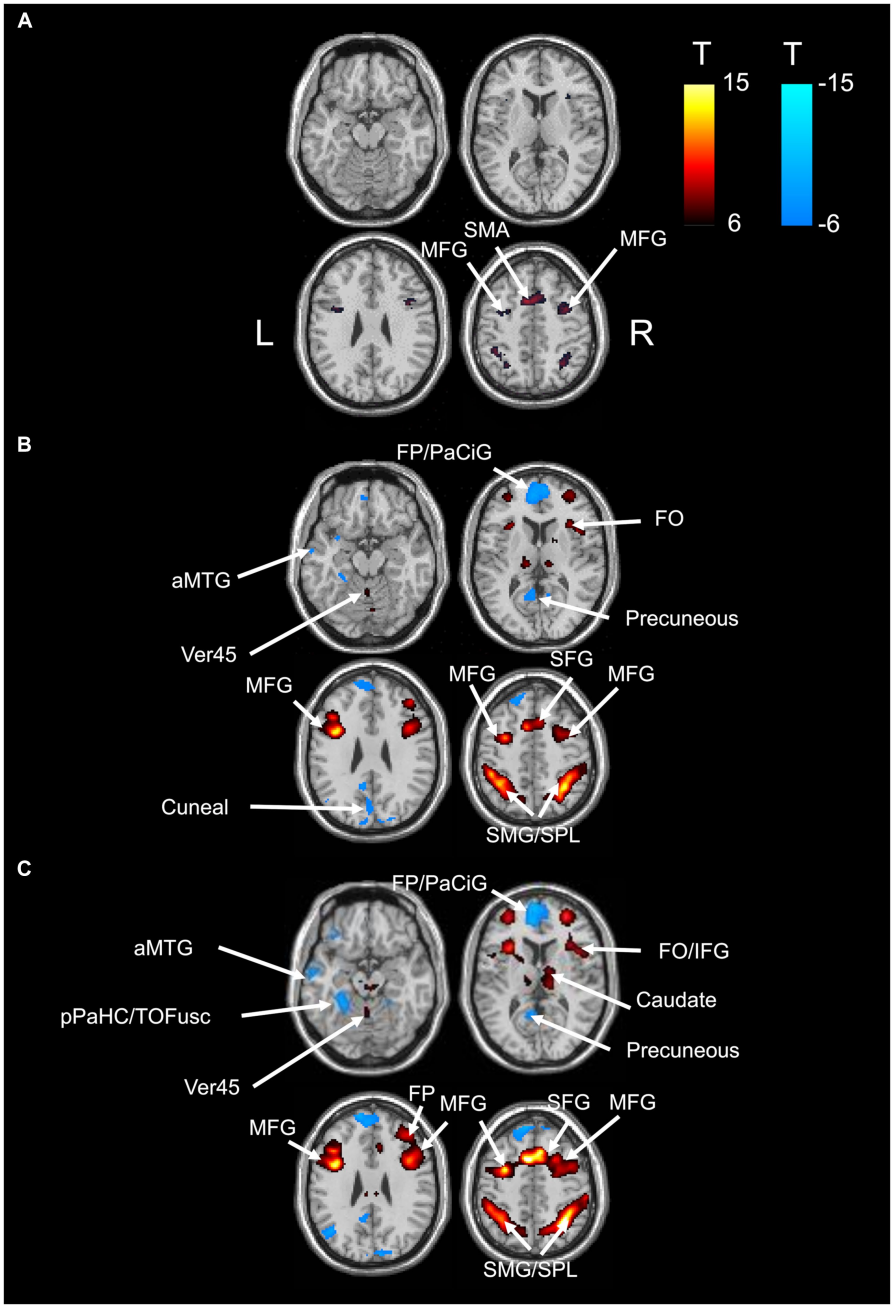
BOLD signal increase during the N-back task. Significant BOLD signal changes in the **(A)** 1-back, **(B)** 2-back, and **(C)** 3-back tasks are shown. The color bar indicates the *t*-value. aMTG, middle temporal gyrus, anterior division; FO, frontal operculum cortex; FP, frontal pole; IFG, inferior frontal gyrus; MFG, middle frontal gyrus; PaCiG, paracingulate gyrus; SFG, superior frontal gyrus; SMG, supramarginal gyrus; SPL, superior parietal lobule; Ver45, vermis 4 5.

BOLD responses were observed in the bilateral MFG and bilateral SFG in all n-back tasks (Figure 4 and Tables 3–7). Positive BOLD responses were observed in the bilateral superior parietal lobule (SPL), bilateral supramarginal gyrus, posterior division, bilateral sLOC, bilateral frontal operculum cortex, bilateral vermis 4 5, right inferior frontal gyrus, pars opercularis, and right angular gyrus in the 2-back and 3-back tasks, but not in the 1-back task. Positive BOLD responses were observed only in the right thalamus and bilateral caudates in the 3-back task. A negative BOLD response in the bilateral FP, bilateral precuneus cortex, and left paracingulate gyrus (PaCiG) was observed in the 2-back and 3-back tasks; however, BOLD signals in no regions were negatively changed in the 1-back task. Negative BOLD responses were only observed in the left parahippocampal gyrus, posterior division, and hippocampus during the 3-back task.

**Table 3:** The significant BOLD increase during 1-back task.

Region	*T*-value	K (mm^3^)	MNI coordinate (mm)		
			x	y	z
Right					
Superior frontal gyrus	9.30	9,970	4	17	68
Middle frontal gyrus	8.71	22,632	45	11	15
Left					
Superior frontal gyrus	10.15	15,920	−3	13	70
Middle frontal gyrus	7.75	11,408	−40	5	38

*T*-value indicates the peak-level *t*-value. *K* indicates the cluster size.

**Table 4:** The significant BOLD increase during 2-back task.

Region	*T*-value	K (mm^3^)	MNI coordinate (mm)		
			x	y	z
Right					
Superior frontal gyrus	8.66	23,240	8	24	65
Middle frontal gyrus	9.92	65,800	48	16	44
Inferior frontal gyrus, pars opercularis	8.44	11,840	55	15	26
Superior parietal lobule	13.24	30,176	39	−55	63
Supramarginal gyrus, posterior division	11.55	14,712	44	−45	55
Angular gyrus	11.05	10,968	45	−46	54
Superior lateral occipital cortex	13.22	35,160	54	69	136
Frontal operculum cortex	11.33	3,256	35	24	12
Left					
Superior frontal gyrus	11.37	19,920	−7	15	67
Middle frontal gyrus	13.51	62,808	−44	10	43
Superior parietal lobule	15.24	41,792	−41	−51	59
Supramarginal gyrus, posterior division	13.29	7,584	−44	−50	58
Superior lateral occipital cortex	11.81	38,056	−32	−58	60
Frontal operculum cortex	11.00	3,880	−33	29	9
Bilateral					
Vermis 4 5	7.02	5,624	−2	−57	−15

*T*-value indicates the peak-level *t*-value. *K* indicates the cluster size.

**Table 5:** The significant BOLD decrease during 2-back task.

Region	*T*-value	K (mm^3^)	MNI coordinate (mm)		
			x	y	z
Right					
Frontal pole	9.09	46,896	10	198	87
Left					
Frontal pole	11.64	61,024	−9	74	3
Anterior middle temporal gyrus	6.94	2072	−65	−4	−15
Paracingulate gyrus	7.93	12,888	−10	55	5
Cuneal cortex	6.85	5,976	−8	−78	30
Bilateral					
Precuneous cortex	8.08	35,880	−9	−52	46

*T*-value indicates the peak-level *t*-value. *K* indicates the cluster size.

**Table 6:** The significant BOLD increase during 3-back task.

Region	T-value	K (mm^3^)	MNI coordinate (mm)		
			x	y	z
Right					
Frontal pole	11.94	39,480	30	60	14
Superior frontal gyrus	14.46	58,384	8	27	62
Middle frontal gyrus	12.03	83,232	50	10	52
Inferior frontal gyrus, pars opercularis	9.81	16,864	55	13	31
Superior parietal lobule	15.12	29,064	41	−54	62
Supramarginal gyrus, posterior division	11.43	13,072	48	−44	58
Angular gyrus	11.92	11,384	42	−53	57
Superior lateral occipital cortex	16.04	44,368	37	−59	62
Frontal operculum cortex	9.58	6,520	39	25	9
Thalamus	8.33	12,520	14	−21	19
Caudate	7.81	4,368	13	−5	21
Left					
Superior frontal gyrus	17.15	39,168	−6	18	65
Middle frontal gyrus	14.07	78,800	−45	11	42
Inferior frontal gyrus, pars opercularis	9.61	8,080	−45	20	11
Superior parietal lobule	12.07	37,976	−40	−52	60
Supramarginal gyrus, posterior division	11.82	7,952	−49	−44	57
Superior lateral occipital cortex	10.75	38,864	−34	−59	60
Frontal operculum cortex	11.24	6,744	−33	29	9
Caudate	6.60	1784	−17	9	17
Bilateral					
Vermis 4 5	6.85	84	0	−59	−9

*T*-value indicates the peak-level *t*-value. *K* indicates the cluster size.

**Table 7:** The significant BOLD decrease during 3-back task.

Region	T-value	K (mm^3^)	MNI coordinate (mm)		
			x	y	z
Right					
Frontal pole	8.31	39,856	4	65	29
Paracingulate gyrus	7.35	6,000	1	55	2
Left					
Frontal pole	11.41	60,704	−6	65	28
Anterior middle temporal gyrus	11.09	8,336	−62	−3	−15
Paracingulate gyrus	10.37	15,672	−5	54	17
Parahippocampal gyrus, posterior division	10.47	1,296	−32	−40	−9
Temporal occipital fusiform cortex	8.91	3,992	−28	−49	−8
Hippocampus	7.54	2,744	−34	−36	−9
Bilateral					
Precuneous cortex	6.85	22,472	−8	−58	23

*T*-value indicates the peak-level *t*-value. K indicates the cluster size.

We compared the areas of significant change in the BOLD responses for the 1-, 2-, and 3-back tasks. The areas of BOLD signal increase and decrease were the largest for the 3-back task (Table 8).

**Table 8:** Cluster size of the significant change in each task.

Task name		Voxel size	T-value threshold
1-back	Increase	1811	6.2
	Decrease	16	6.2
2-back	Increase	10,757	6.0
	Decrease	3,442	6.0
3-back	Increase	15,377	6.1
	Decrease	3,999	6.1

The correlation between the fMRI beta value of the bilateral MFG and SFG, which were activated in all N-back tasks, and task accuracy was investigated. In 1-back and 3-back task had small *R*^2^ in these ROIs. The *R*^2^ in 1-back was 3.42 × 10^−2^ for right SFG, 4.66 × 10^−5^ for left SFG, 8.56× 10^−5^ for right MFG, and 5.27 × 10^−3^ for left MFG. The *R*^2^ in 3-back was 3.61 × 10^−2^ for right SFG, 6.13 × 10^−2^ for left SFG, 4.96 × 10^−2^ for right MFG, and 6.04 × 10^−2^ for left MFG. The 2-back task showed weak correlation in left MFG (*R*^2^ = 1.93 × 10^−1^), left SFG (*R*^2^ = 2.89 × 10^−1^) and left MFG (*R*^2^ = 2.64 × 10^−1^) and very weak correlation in right SFG (*R*^2^ = 9.01 × 10^−3^). These results indicate that there is no significant correlation between BOLD response and task performance (with an R^2^ > 0.3).

### Correlation between structural/functional connectivity and accuracy during the N-back task

From the behavioral analysis, we used the accuracy of each N-back task to investigate the correlation between behavior and structural connectivity (Table 9). For 1-back task, a connection between cerebellum 3 and right supplementary motor area showed higher correlation (*R*^2^ > 0.4), and no connection had *R*^2^ > 0.5. There is highest correlation for 2-back task in n-back task (10 connections had *R*^2^ > 0.4, and 2 connections had *R*^2^ > 0.5). In contrast, 3-back task showed no connection that had *R*^2^ > 0.4. Notably, several structural connections had the correlation with accuracy in 1-back and 2-back tasks, i.e., left cerebellum 3 and right SMA, Vermis 3 and right SFG, right SPL and right MFG, and right frontal orbital cortex and right inferior frontal gyrus, pars triangularis. However, it should be noted that R^2^ for the accuracy of 1-back task was lower than that of 2-back task. Furthermore, a correlation was found between structural connectivity and accuracy in several brain regions activated during the 2-back task, such as the bilateral SPL, right SMA, right angular gyrus, right SFG, right MFG, and left thalamus.

**Table 9:** The top 10 structural connectivity in each task.

Connectivity		R^2
1-back		
Left cerebelum 3	Right supplementary motor area	0.40
Vermis 3	Right superior frontal gyrus	0.34
Left thalamus	Cingulate Gyrus, anterior division	0.33
Left superior frontal gyrus	Left lateral occipital cortex, superior division	0.28
Right superior parietal lobule	Right middle frontal gyrus	0.28
Right Cerebelum 10	Right Pallidum	0.28
Right Cerebelum 8	Left Cerebelum 8	0.27
Left accumbens	Left occipital fusiform gyrus	0.27
Right frontal orbital cortex	Right inferior frontal gyrus, pars triangularis	0.27
Right middle frontal gyrus	Right inferior frontal gyrus, pars opercularis	0.26
2-back		
Left cerebelum 3	Right supplementary motor area	0.54
Left thalamus	Right superior parietal lobule	0.52
Right angular gyrus	Right superior frontal gyrus	0.47
Right superior lateral occipital cortex	Left superior parietal lobule	0.45
Left planum temporale	Left inferior frontal gyrus, pars triangularis	0.44
Right angular gyrus	Right middle frontal gyrus	0.43
Right frontal orbital cortex	Right inferior frontal gyrus, pars triangularis	0.42
Right superior parietal lobule	Right middle frontal gyrus	0.41
Right superior lateral occipital cortex	Right middle frontal gyrus	0.40
Vermis 3	Right superior frontal gyrus	0.40
3-back		
Right amygdala	Right precentral gyrus	0.38
Right angular gyrus	Right precentral gyrus	0.38
Left cerebelum crus1	Right superior frontal gyrus	0.36
Vermis 3	Left cerebelum crus2	0.35
Vermis 8	Left cerebelum 3	0.35
Right putamen	Right intracalcarine cortex	0.35
Right Heschl’s gyrus	Precuneous cortex	0.35
Left cerebelum 7b	Right pallidum	0.34
Vermis 7	Vermis 3	0.33
Left cerebelum 8	Right superior frontal gyrus	0.33

The simple regression analysis between functional connectivity and 2-back and 3-back task accuracies were also performed, but there was no functional connectivity with an *R*^2^ > 0.3.

## Discussion

In the present study, we compared the local activation, functional connectivity, and structural connectivity associated with the N-back task in the same participants. Functional connectivity during the N-back task was altered in a manner that differed according to the difficulty of the N-back task. In contrast, GLM analysis revealed that the area of significant BOLD response during the N-back task increased in the 3-back task compared to that in the 1- and 2-back tasks, but the location of local activation was similar between the 2-back and 3-back tasks, except for the thalamus and caudate. The differing results between GLM and gPPI analyses can be explained by information on BOLD signals. GLM analysis detected the local BOLD response related to neuronal activation during the N-back task compared with the resting period. In contrast, gPPI analysis detects functional connectivity, which is the synchronization of neuronal fluctuations influenced by the N-back task. A previous study revealed that local activation is not necessarily related to connectivity changes in large-scale brain networks, which is consistent with the results of the present study (Gerchen and Kirsch, 2017). The structural connectivity associated with the accuracy of the 2-back task involved the regions activated during the 2-back task (R^2^ > 0.3). However, increased functional connectivity using gPPI analysis and local activation did not show a significant correlation with accuracy. Previous study showed the weak correlation between local activation and accuracy (R^2^ < 0.3), corresponding to current study (Lamichhane et al., 2020). The structural connectivity refers to the white matter pathways between regions, while functional connectivity and local activation refer to the node-to-node interactions and local activity between neurophysiologically active regions. These results indicate that task performance is related to the white matter pathways rather than the neuronal activity. Another study also shows that the training changes the white matter structure, indicating the relationship between white matter structure and task performance (Scholz et al., 2009).

Functional connectivity significantly differed between the 2-back and 3-back tasks, whereas it did not change significantly in the 1-back task (Figure 3A and Table 1). In the 2-back task, functional connectivity increased in the regions related to word perception and working memory. The inferior temporal cortex, including the ITG, temporo-occipital region, and aSMG, is essential for language comprehension and cognition (Kourtidou et al., 2022). The temporal cortex, including the left TP and right aMTG, is associated with word meaning (Mesulam et al., 2013). The posterior cingulate cortex is essential for working memory (Leech and Sharp, 2014). The visual cortex, which is related to word processing, was negatively correlated with several regions of the temporal cortex (left TP, right aMTG, left parahippocampal gyrus, and posterior division). Notably, most brain regions that showed increased functional connectivity were not locally activated (Figure 4), indicating a different manner of neuronal activity between the GLM and gPPI analyses. In contrast, the 3-back task increased the functional connectivity in regions mainly related to verbal processing and motor regulation (Figure 3B and Table 2). Functional connectivity was increased between the insular cortex/operculum, motor cortex, hippocampus, and basal ganglia. Although the operculum and the insular cortex are essential for the N-back task (Namkung et al., 2017), other regions are mainly involved in motor regulation (Lanciego et al., 2012). Functional connectivity between the occipital lobe, which is related to verbal processing, and the hippocampus was decreased in the 3-back task. Furthermore, functional connectivity decreased between the frontal lobe and basal ganglia, which are related to perception and motion control, in the 3-back task. This distinct functional connectivity in the N-back task may be due to the difficulty of the working memory task. Despite the high accuracy for all volunteers, the response time depended on the individual for the 1-back task, indicating that it was easy to perform for all participants. The response time and accuracy varied widely for the 3-back task, indicating that the 3-back task exceeded the working memory capacity of some volunteers. A weak correlation was found between accuracy and response time for the 2-back task. Therefore, we decided that the 2-back task is better to investigate working memory than the 1-, and 3-back tasks. Importantly, the increased functional connectivity for the 3-back task did not include the regions essential for working memory and word meaning, indicating that the 3-back task exceeded the capacity of working memory in some volunteers. The present study also shows the correlation between accuracy and local activations was higher in 2-back task than in 1- and 3-back tasks. This implies that the brain uses primitive brain networks related to intuitive decisions in complex working memory tests.

As described above, the local activation and functional connectivity are triggered by neuronal activation (Drew, 2019) and neuronal oscillation (Takeuchi et al., 2010) influenced by the task. A previous study revealed that local activation is not necessarily similar to connectivity changes in brain networks, which supports the results of the present study (Gerchen and Kirsch, 2017). Functional connectivity is related to the synchronization of neuronal oscillations. A previous study has revealed that local brain activation and task-dependent connectivity changes can be modulated in different directions during motor tasks. Local activation and functional connectivity are two aspects of the laterality of single-hand movements in different manner (Tsurugizawa et al., 2023). These results indicate that a combination of GLM (local activation) and gPPI (functional connectivity) analyses is better suited for investigating the neuronal mechanisms of working memory.

The top 10 structural connectivity associated with accuracy in the 2-back task were locally activated during the 2-back task. Structural connectivity with the right MFG, inferior frontal gyrus, and SPL correlated with accuracy in the 2-back task, and these regions were activated during the 1- and 2-back task. However, the R^2^ in 1-back task is smaller than 2-back. Because the SFG and MFG showed increased functional connectivity, as well as local activation and structural connectivity, these regions may be key regions for the N-back task. A previous study showed that the clustering coefficients of the right anterior cingulate gyrus and right inferior ventrolateral prefrontal cortex significantly increased from pre-training to post-training in a learning memory task (Caeyenberghs et al., 2016). The structural connectivity that identifies white matter bundles and connects the nodes of functional networks reflects the physical connections between anatomically separated regions. This physical network contributes to the local activation during working memory tasks. A previous study has revealed that structural connectivity is affected by training and working memory (Rogers et al., 2007). Structural connectivity predicts local activation, suggesting the increased sensitivity of this technique in identifying individual differences in task-based fMRI activation (Ekstrand et al., 2020). These results indicate that structural connectivity, rather than functional connectivity, is useful for predicting local activation. Notably, the observed functional connectivity for the 2-back task did not correspond to structural connectivity. Instead, previous study shows that resting-state functional connectivity can be predicted from structural connectivity (Chen and Wang, 2018). However, altered functional connectivity during working memory tasks is not linked to structural connectivity. This indicates that functional connectivity changes during the task provide distinct information compared with structural connectivity and BOLD responses. Previous studies have reported that resting state functional connectivity in several brain regions increases following training for working memory (Cole et al., 2021; Pongpipat et al., 2021; Yeung and Han, 2023) and structural connectivity (Roman et al., 2017). The default mode network also decreases when the working memory load increases (Ginestet and Simmons, 2011). As recent study indicates, the combination of structural and functional connectivity can be better predictor of working memory accuracy (Kim et al., 2021). As the current study investigated functional connectivity in naïve participants, future studies should investigate dynamic changes in structural and functional connectivity following working memory training to assess as the predictor of working memory accuracy.

In conclusion, both GLM (local activation) and gPPI (functional connectivity) analyses revealed a new aspect of working memory. Structural connectivity is useful for predicting working memory accuracy, although functional connectivity and local activation shows weak correlation with it. Therefore, using a comprehensive approach of fMRI and DTI is better for gaining an understanding of the neuronal mechanisms of working memory.

### Limitation of the study

Large sample sizes have recently been required to obtain reliable results in fMRI studies, and meta-analyses using this database have been performed for N-back tasks (Owen et al., 2005; Yaple et al., 2019). Although the brain region of local activation observed during the N-back task is consistent with these studies, and the difficulty-dependent increase in the area of local activation has also been reported in previous studies (Lamichhane et al., 2020), the results of the current study should be investigated with a larger sample size in the future.

In the present study, 1, 2, 3-back tasks and no N-back task were conducted in different sessions. Therefore, it was not possible to compare the fMRI response in each n-back task directly. In the future, this analysis should be assessed with the data sets that includes the fMRI data of no-back, 1, 2, and 3-back tasks in the same run for the direct comparison.

## Data availability statement

The raw data supporting the conclusions of this article will be made available by the authors, without undue reservation.

## Ethics statement

The studies involving humans were approved by Institutional Review Board of the National Institute of Advanced Industrial Science and Technology. The studies were conducted in accordance with the local legislation and institutional requirements. The participants provided their written informed consent to participate in this study.

## Author contributions

TS: Investigation, Validation, Visualization, Writing – original draft, Writing – review & editing. AT: Investigation, Writing – review & editing. KK: Methodology, Writing – review & editing. DY: Methodology, Writing – review & editing. TT: Conceptualization, Funding acquisition, Investigation, Methodology, Project administration, Supervision, Validation, Visualization, Writing – original draft, Writing – review & editing.

## Footnotes

^1^https://www.psychopy.org/

^2^www.Conn-toolbox.org
